# Downregulation of CXCR4 in Metastasized Breast Cancer Cells and Implication in Their Dormancy

**DOI:** 10.1371/journal.pone.0130032

**Published:** 2015-06-17

**Authors:** Kentaro Nobutani, Yohei Shimono, Kiyohito Mizutani, Yuki Ueda, Toshihiro Suzuki, Midori Kitayama, Akihiro Minami, Kenji Momose, Kohta Miyawaki, Koichi Akashi, Takeshi Azuma, Yoshimi Takai

**Affiliations:** 1 Division of Pathogenetic Signaling, Department of Biochemistry and Molecular Biology, Kobe University Graduate School of Medicine, Kobe, Hyogo, Japan; 2 Division of Gastroenterology, Department of Internal Medicine, Kobe University Graduate School of Medicine, Kobe, Hyogo, Japan; 3 Division of Molecular and Cellular Biology, Department of Biochemistry and Molecular Biology, Kobe University Graduate School of Medicine, Kobe, Hyogo, Japan; 4 Department of Oral and Maxillofacial Surgery, Kobe University Graduate School of Medicine, Kobe, Hyogo, Japan; 5 Department of Medicine and Biosystemic Science, Kyushu University Graduate School of Medical Sciences, Fukuoka, Fukuoka, Japan; Florida International University, UNITED STATES

## Abstract

Our understanding of the mechanism of cancer dormancy is emerging, but the underlying mechanisms are not fully understood. Here we analyzed mouse xenograft tumors derived from human breast cancer tissue and the human breast cancer cell line MDA-MB-231 to identify the molecules associated with cancer dormancy. In immunohistological examination using the proliferation marker Ki-67, the tumors included both proliferating and dormant cancer cells, but the number of dormant cells was remarkably increased when they metastasized to the lung. In the gene expression analysis of the orthotopic cancer cells by a single-cell multiplex real-time quantitative reverse transcription PCR followed by flow cytometric analysis, restrained cellular proliferation was associated with downregulation of the chemokine receptor CXCR4. In the immunohistological and flow cytometric analyses, the expression level of CXCR4 in the metastasized cancer cells was decreased compared with that in the cancer cells in orthotopic tumors, although the expression level of the CXCR4 ligand CXCL12 was not reduced in the lung. In addition, the proliferation of the metastasized cancer cells was further decreased by the CXCR4 antagonist administration. In the *ex vivo* culture of the metastasized cancer cells, the expression level of CXCR4 was increased, and in the xenotransplantation of *ex vivo* cultured cancer cells, the expression level of CXCR4 was again decreased in the metastasized cancer cells in the lung. These findings indicate that CXCR4 is downregulated in metastasized breast cancer cells and implicated in their dormancy.

## Introduction

Cancer dormancy is a phenomenon that allows cancer cells long-term survival and resistance to cancer therapies [1]. This process involves multiple biological factors, such as immunological adaptation, angiogenesis, cell adhesion, and stemness of cancer cells [1,2]. Dormant cancer cells survive even in metastasized organs and cause late relapse of the disease after a successful cancer treatment [2]. Clinical evidence suggests that metastasized dormant cancer cells exit the dormant state when extracellular conditions and intrinsic cellular characteristics become more favorable for their growth [3]. Recent studies using mouse models of cancer metastasis have revealed the extrinsic and intrinsic factors that are associated with the induction and maintenance of cancer dormancy. Cancer dormancy is induced in metastasized cancer cells by extrinsic factors, such as bone morphologic protein, thrombospondin-1, and TGF-β2, which are derived from the tissues where the cancer cells metastasized [4–6]. Regarding the intrinsic factors, the activity of the extracellular signal-regulated kinase signaling is decreased, whereas the p38 signaling activity is increased in dormant cancer cells [7]. However, the mechanisms for the induction, maintenance, and exit of cancer dormancy are still unclear.

We recently generated breast cancer tumor models in mice by orthotopic xenotransplantation of human breast cancer tissues obtained from breast cancer patients and the human breast cancer cell line MDA-MB-231 [8,9]. Using these models, we analyzed the relationship between cell proliferation and the presence of primary cilia in cancer cells using the cell proliferation marker Ki-67 [9]. Cancer cells in these models showed heterogeneity in terms of proliferating activity, and Ki-67-positive proliferating cancer cells were dominant in the orthotopic tumor. In contrast, the cancer cells that spontaneously metastasized to the lung in the early course of the disease stayed in the organ with more restrained proliferating activity than in the orthotopic tumor, at least in part, representing the induction of the dormant state of the cancer cells. Consistent with previous reports [4–6,10], our results suggest that not all cancer cells uniformly proliferate in the tumor and metastatic tissues, and that instead they putatively change proliferating activity depending on the change of the environment that allows the cancer cells to enter to or exit from dormancy.

In this study, we analyzed breast cancer cells in mouse xenograft tumors to identify the intrinsic factors that are associated with cancer cell dormancy. Single-cell multiplex gene expression analysis of the xenograft tumors revealed the downregulation of the chemokine receptor CXCR4 in the dormant cancer cells. CXCR4 is a member of the C-X-C chemokine receptor family that is associated with a wide range of biological processes, such as hematopoiesis [11], development of organs [11], inflammatory responses [12], cell survival [13], and G0/G1 transition [14]. Furthermore, CXCR4 is involved in various aspects of cancers, such as metastasis [15], tumor growth [16], cell cycle progression [17], and drug resistance [18]. We then applied this finding to the dormancy of metastasized breast cancer cells and found dynamic changes of the expression level of CXCR4 in cancer cells along with the entrance and exit of cancer cell dormancy.

## Materials and Methods

### Cell Culture

MDA-MB-231 cells [8] were maintained in Dulbecco’s modified Eagle’s medium (DMEM) supplemented with 10% FBS, 100 U/ml penicillin, and 100 μg/ml streptomycin, and cultured at 5% CO_2_ at 37°C.

### Generation of xenograft tumor-bearing mice

All animal experiments were performed under the approval of the Kobe University Animal Care and Use Committee (Permission number: P100905) and carried out according to the Kobe University Animal Experimentation Regulation. All surgical procedures were performed under isoflurane anesthesia with care to minimize suffering of mice. Female non-obese diabetic severe combined immunodeficiency (NOD-SCID) mice were purchased from CLEA and maintained in the animal facility of Kobe University Graduate School of Medicine. The breast cancer tissue samples were collected with written informed consent from surgically resected breast cancer tissues of the breast cancer patients who were admitted to Kobe University Hospital as approved by the Research Ethics Board at Kobe University Graduate School of Medicine (Permission number: 1481). The tissues were minced and suspended in Matrigel (BD Biosciences), and then transplanted into the inguinal mammary fat pad regions of NOD-SCID mice as previously described [19]. Four patient-derived xenograft (PDX) lines were established with this method as previously reported [9]. In the generation of the cell line-derived xenograft tumor, MDA-MB-231 cells were infected with a lentivirus vector encoding ZsGreen1 (Clontech), and ZsGreen1-expressing cells were sorted by FACS Aria I (BD Biosciences) and cultured in DMEM supplemented with 10% FBS, 100 U/ml penicillin, and 100 μg/ml streptomycin at 5% CO_2_ at 37°C. Then, 4 × 10^6^ of the ZsGreen1-expressing cells were suspended in Matrigel and transplanted into the inguinal mammary fat pad regions of NOD-SCID mice. When the tumor reached approximately 1–2 cm in size in about 1.5–3 months, the orthotopic tumors and the lung tissues were analyzed.

### Antibodies

An Alexa Fluor 488-conjugated mouse anti-human leukocyte antigen (HLA)-A, B, C monoclonal Ab (mAb) (clone: W6/32) was purchased from BioLegend. A rabbit anti-human Ki-67 (H-300) polyclonal Ab (pAb) was purchased from Santa Cruz Biotechnology. A rat anti-mouse CD31 mAb (clone: MEC13.3) and an allophycocyanin-conjugated mouse anti-human CD184 mAb (clone: 12G5) were purchased from BD Biosciences. A mouse anti-human CXCR4 mAb was purchased from Abcam (clone: 44716.111). A mouse anti-CXCL12 mAb was purchased from Millipore (clone: K15C). An Alexa Fluor 555-conjugated goat secondary Ab was purchased from Invitrogen.

### Immunohistological examinations

For the immunofluorescence staining of Ki-67 and CXCR4, the orthotopic xenograft tumor and the lung tissues were directly frozen in O.C.T. Compound (Sakura Finetek). For the immunofluorescence staining of CD31, the tissues were fixed overnight in 2% paraformaldehyde/phosphate buffered saline (PBS) solution at 4°C and then frozen in O.C.T Compound. The frozen tissues were sliced into 10-μm-thick sections using a cryostat, and the following procedures were performed at room temperature. For the staining of Ki-67, the sliced sections were fixed in acetone for 5 min, and for the staining of CXCR4, the sliced sections were fixed in 4% paraformaldehyde/PBS solution for 1 h. For the staining of CD31, the sections were incubated with 0.1% Triton X-100/PBS solution for 10 min, and for the staining of CXCR4, the sections were incubated with 0.1% saponin/PBS solution for 10 min. For the staining of CXCL12, the tissues were fixed 3 h in Bouin’s fixative solution at 4°C, washed in ethanol three times, and embedded in paraffin. The sections of paraffin-embedded specimens were deparaffinized in xylene (twice) and treated with a graded series of alcohol (100%, 95%, and 80% ethanol/double-distilled water (v/v)), rehydrated in PBS, and were subjected to the antigen retrieval technique using HistoVT One (Nacalai Tesque) as described in the manufacturer's protocol. The sections were washed with 10 mM glycine/PBS solution, and then blocked with 3% bovine serum albumin for 30 min. After another wash with PBS, the sections were incubated with appropriate Abs for 2 h and then incubated with the corresponding secondary Ab and DAPI for 1 h (dilutions: CD31, 1:200; Ki-67, 1:100; CXCR4, 1:200; CXCL12, 1:35; secondary Ab, 1:200; DAPI, 1:400). For the staining of HLA, the specimens were incubated with an Alexa Fluor 488-conjugated mouse anti-HLA-A, B, C mAb for 1 h (dilution: 1:100). The sections were observed using a confocal laser scanning microscope (LSM 700, Carl Zeiss, or C2, Nikon). The number of the Ki-67-positive or negative cancer cells in the orthotopic tumors and the lung were manually counted.

### Flow cytometric analysis

The xenograft tumors and the lung tissues were dissociated using collagenase I and III (Worthington) by a previously described method [20]. The cultured cells were detached from the bottom of the culture dish using 0.5% Trypsin–EDTA (Nacalai Tesque). The cells were stained with an allophycocyanin-conjugated mouse anti-human CD184 mAb, and dead cells were excluded by staining with 7-amino-actinomycin D (BD Biosciences). The cells of the dissociated PDX stained with Alexa Fluor 488-conjugated mouse anti-human HLA-A, B, C mAb and the cell line-derived tumor cells expressing ZsGreen1 were analyzed and sorted using FACS Aria I (BD Biosciences). In the cell cycle analysis, the sorted cells were subsequently permeabilized by a detergent solution (0.5% Triton-X 100 in PBS, 0.5 mM EDTA, pH 7.2), and then stained with propidium iodide (Sigma) with addition of RNase (Sigma) as previously described [21]. In the *ex vivo* culture experiment, the high expression level of cell surface CXCR4 was determined as the signal level that includes more than 50% of the orthotopic cancer cells and less than 5% of the metastasized cancer cells in the lung on average.

### Administration of AMD3100

AMD3100 (Sigma) was diluted with PBS to a concentration of 1 mg/ml. To analyze the effect of AMD3100 on the dormancy of the metastasized cancer cells in the lung, the tumor at the orthotopic site was surgically removed 6 weeks after the transplantation of MDA-MB-231 cells, when the tumor metastasized to the lung. One week after the surgical removal, AMD3100 solution containing 100 μg of AMD3100 or the vehicle solution was administered by daily subcutaneous injection for 2 weeks. To analyze the effect of AMD3100 on the proliferation of cancer cells at the orthotopic site, AMD3100 was administered at the lesion around the orthotopic xenograft tumors every 2–3 days until 25 days after the xenotransplantation of the MDA-MB-231. The tumor size was recorded every time before administration of the solutions.

### Real-time quantitative reverse transcription PCR

For the single-cell gene expression analysis, the cancer cells in the orthotopic xenograft tumor were sorted to 96-well PCR plates, and multiplex real-time quantitative reverse transcription PCR (RT-PCR) in which RT was followed by 40-cycles of PCR was performed using Cells Direct One-Step qRT-PCR Kits (Life Technologies), TaqMan Gene Expression Assays (Life Technologies), and Biomark HD (Fluidigm) as described in the manufacturer's protocols. In the analysis of the expression level of the tissue *Cxcl12* mRNA, pieces of tissues (about 8 mm^3^ in size) were obtained from orthotopic tumors and lungs. Tissues were homogenized in 1 ml Trizol Reagent (Invitrogen), and RNA was extracted from the homogenates according to the manufacturer’s instructions. The RNA samples were subjected to the real-time quantitative RT-PCR using a High Capacity cDNA Reverse Transcription Kit (Life Technologies), TaqMan Gene Expression Assays (*Gapdh* and *Cxcl12*, Life Technologies), TaqMan Fast Advanced Master Mix (Life Technologies), and Step One Plus real-time PCR system (Life Technologies) according to the manufacturer’s instructions. Data were normalized to the amount of *Gapdh* cDNA as an endogenous control.

### *Ex vivo* culture of the cancer cells in the tumor-bearing mice

The orthotopic tumors or the lung tissues of the mice bearing MDA-MB-231-derived xenograft tumor were dissociated as described above. One million orthotopic tumor cells and the same number of lung cells that included spontaneously metastasized MDA-MB-231 cells were cultured at 5% CO_2_ at 37°C in 10-cm culture dishes with DMEM supplemented with 10% FBS, 100 U/ml penicillin, and 100 μg/ml streptomycin. Growth of the cancer cells was monitored by fluorescence microscopy. After 5–7 days when the cells became 70–80% confluent, and at 9–13 days when cells became completely confluent, the expression level of CXCR4 in the cancer cells was analyzed by flow cytometry as described above. The cells cultured for 9–13 days were then suspended in Matrigel and xenotransplanted to other NOD-SCID mice. The number of the cancer cells in the culture dish at Day 0 and Day 7 of the *ex vivo* culture was examined using flow cytometry, and the ratio between them was calculated as a proliferation rate of the cancer cells.

### Statistical analysis

The data are reported as means ± SEM. Statistical significance was analyzed using unpaired Student’s t-test for comparison between two groups, and non-repeated measures ANOVA, followed by the Student–Newman–Keuls test for comparison among more than two groups. A P-value less than 0.05 was considered statistically significant.

## Results

### Cancer cell dormancy in the breast cancer xenograft tumor models

**Fig 1:**
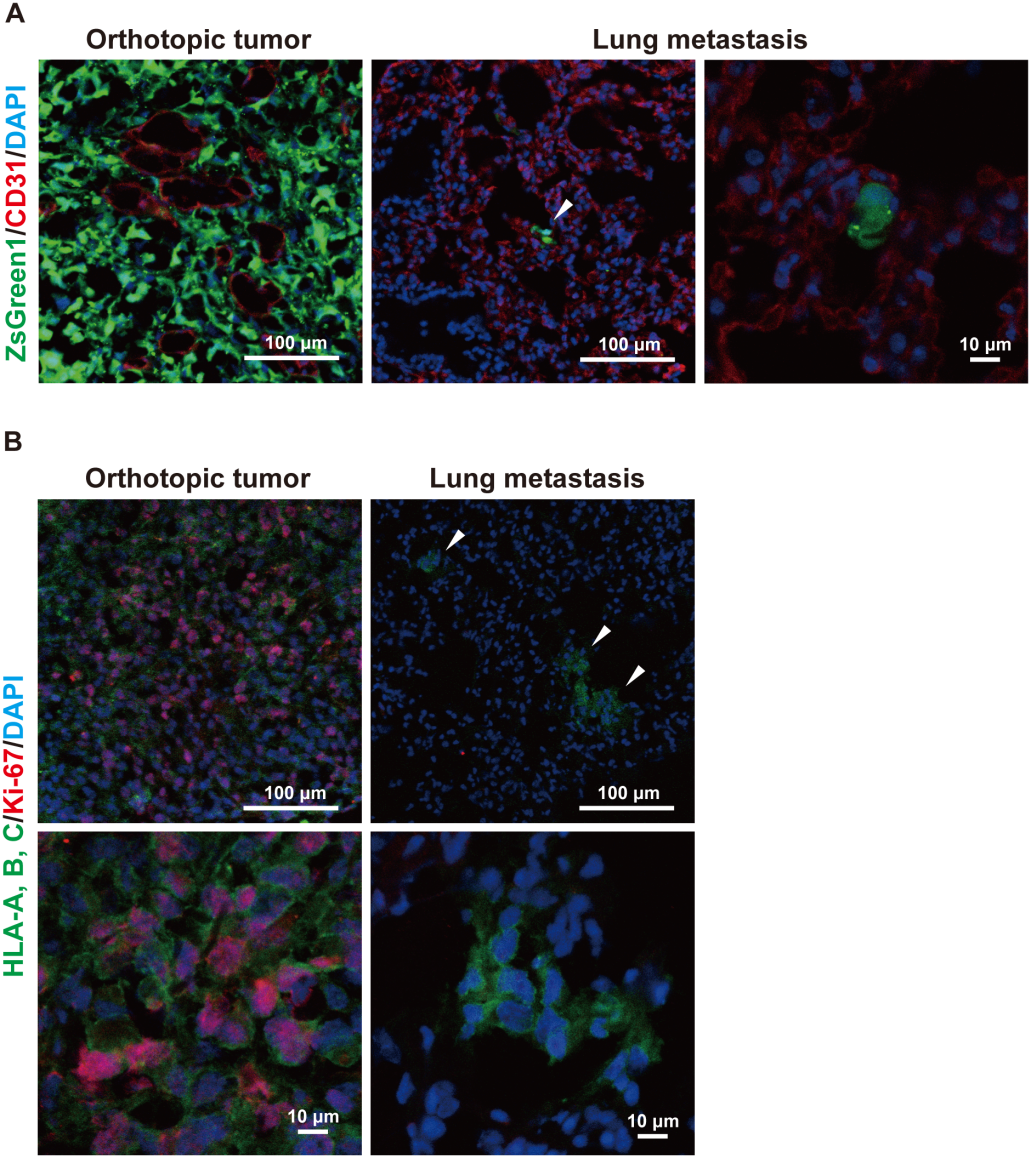
Dormancy of breast cancer cells in the xenograft tumor-bearing mice. (A) Immunofluorescent images for the endothelial cell marker, CD31, and MDA-MB-231 breast cancer cells in the orthotopic tumor and metastatic lesions in the lung. The arrowhead indicates the metastatic tumor. Green: ZsGreen1; red: CD31; blue: nucleus. Scale bars: 100 μm for the low power field; 10 μm for the high power field. Representative images are shown. (B) Immunofluorescent images for Ki-67 in the MDA-MB-231 breast cancer cells in the orthotopic tumor and metastatic lesions in the lung. The arrowheads indicate the metastatic tumor lesions in the lung. Green: human leukocyte antigen (HLA)-A, B, C; red: Ki-67; blue: nucleus. Scale bars: 100 μm for the low power field; 10 μm for the high power field. Representative images are shown.

To examine the characteristics of dormant breast cancer cells, we analyzed xenograft tumors in mice that we generated by orthotopic xenotransplantation of human breast cancer tissues obtained from breast cancer patients and the human breast cancer cell line MDA-MB-231 [9]. In the following experiments, we used one of the PDX lines and one from the cell line that allowed us to detect and isolate the cancer cells by the green fluorescence protein ZsGreen1 that was transduced to the cell line or the expression of the HLA-A, B, C (Figs 1, 3, 4 and 5; S1, S2 and S3 Figs). The human breast cancer PDX was generated from the ER (+)/PR (+)/HER2 (-) breast cancer and the MDA-MB-231 cells showed triple-negative breast cancer phenotype [9,22]. The growth rate of the PDX was much lower than that of the cell line-derived xenografts: the PDX and cell line-derived xenografts reached approximately 1–2 cm in about 3 months and 5–6 weeks, respectively. In the cell line-derived tumor model, when the tumors grew to these sizes, a small number of the cancer cells spontaneously metastasized to the lung and located at the perivascular area (Fig 1A and 1B). The metastatic cancer cells in the lung were also detected by flow cytometry (S1 Fig). Essentially the same results were obtained in the human PDX model (S2A and S2B Fig). In the cell line-derived tumor model, the metastatic cancer cells in the lung were present solely or in small clusters in contrast to the cancer cells in the orthotopic tumors, in which a large number of the cancer cells were densely packed in the tumors (Fig 1A and 1B). More than 80% of the cancer cells in the orthotopic tumors excluding those in the necrotizing tissue were positively stained for anti-Ki-67 pAb, although there was a small fraction of Ki-67-negative cancer cells (Fig 1B and Table 1). In contrast, consistent with a previous report [5], cancer cells not stained with the anti-Ki-67 pAb were dominant in the lung, and less than 20% of the metastasized cancer cells in the lung were Ki-67-positive (Fig 1B and Table 1). The Ki-67-positive normal lung cells were rarely observed (data not shown). Essentially the same results were obtained in the PDX model (S2B Fig and Table 1). Therefore, cancer cells in the orthotopic tumor are prone to be in the proliferating state, whereas the metastasized cancer cells in the lung tend to be in the dormant state. These findings suggest that the proliferating activity of the cancer cells is heterogeneous, and that the number of the proliferating cancer cells changes depending on the change of the tumor environment.

**Table 1:** Numbers and percentages of Ki-67-positive or negative cancer cells.

Tumor origin	Tumor site	Ki-67 (+)*	Ki-67 (-)*	Percentage of Ki-67 (+) cells
MDA-MB-231	Orthotopic tumor	291	38	88
	Lung metastasis	24	261	8
Human tissue	Orthotopic tumor	220	47	82
	Lung metastasis	47	233	17

*Number of the Ki-67-positive or negative cancer cells in the orthotopic tumors and the lung metastases. The comparable numbers of cancer cells in the orthotopic tumor and the lung metastases were analyzed.

### Downregulation of CXCR4 in dormant cancer cells in the breast cancer xenograft tumors

**Fig 2:**
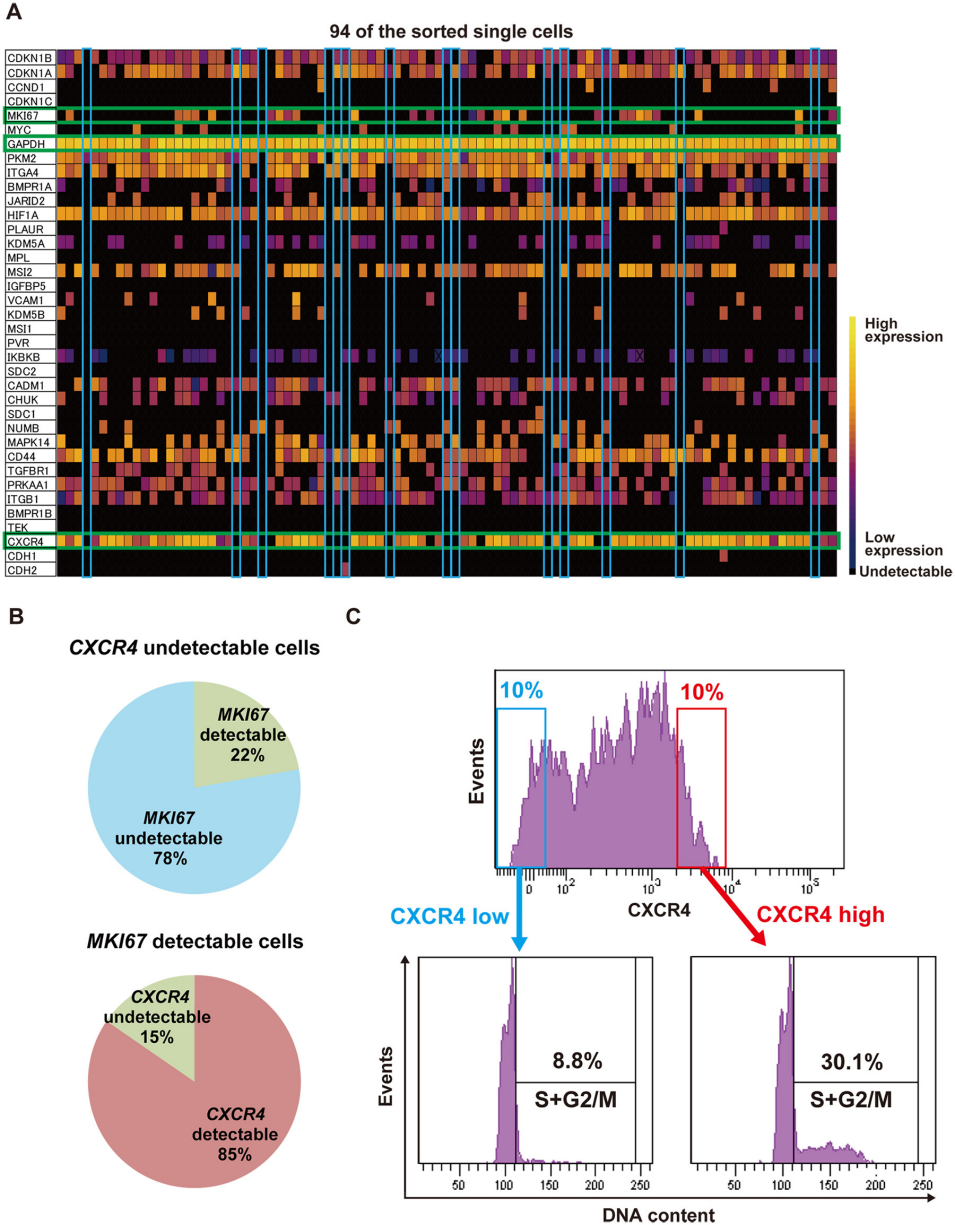
Downregulation of CXCR4 in dormant cancer cells in the human breast cancer patient-derived xenograft (PDX) model. (A) Heat map image of gene expression of 94 single breast cancer cells that were analyzed by the single-cell multiplex real-time quantitative reverse transcription PCR (RT-PCR) for 37 selected genes. Each of the squares filled with color shows the gene expression level of each single cell. Yellow indicates a high expression level, dark blue indicates a low expression level, and black indicates an undetectable level of gene expression. Green squares indicate the expression levels of *MKI67*, *GAPDH*, and *CXCR4*. Sky blue squares indicate cells showing undetectable expression levels of both *MKI67* and *CXCR4*. All cells showed an appropriate expression level of *GAPDH*. (B) Charts showing the relationship between the expression levels of *MIK67* and *CXCR4*. The results of the single-cell real-time quantitative RT-PCR were summarized. (C) Flow cytometric analysis of the expression level of cell surface CXCR4 and cell cycle of the cancer cells in the orthotopic tumors of the human breast cancer PDX. Representative histograms are shown.

To identify the intrinsic factors associated with the heterogeneity and dynamic change of growth activity, we performed single cell sorting of approximately 100 cancer cells using the orthotopic tumor of the PDX model to analyze the gene expression profiles of proliferating and dormant cancer cells. We performed the single-cell multiplex real-time quantitative RT-PCR analyses for 37 genes that were selected based on association with cell proliferation, cell adhesion, metabolism, epigenetic regulation, signal transduction, and stemness. The 94 cells that showed appropriate expression levels of housekeeping genes were subjected to the analysis. The heat map that showed the relative expression level of each gene in a single sorted cell revealed a relationship between the expression levels of *MKI67*, a gene encoding the marker of proliferation Ki-67, and *CXCR4* (Fig 2A). Most cells with an undetectable expression level of *CXCR4* expressed an undetectable level of *MKI67* whereas most cells with a detectable expression level of *MKI67* expressed a detectable level of *CXCR4* (Fig 2A and 2B). To validate this finding, 10% of the total cells expressing either a high or low level of CXCR4 were sorted from the tumor, and the cell cycle of each group was analyzed (Fig 2C). The percentage of cells in the proliferating stage was remarkably lower in cells with a lower expression level of CXCR4 in contrast to the cells with a higher expression level of CXCR4 (Fig 2C). These findings show a possible molecular link between the low expression level of CXCR4 and the suppression of cell proliferation in the cancer cells in the human breast cancer PDX in mice.

**Fig 3:**
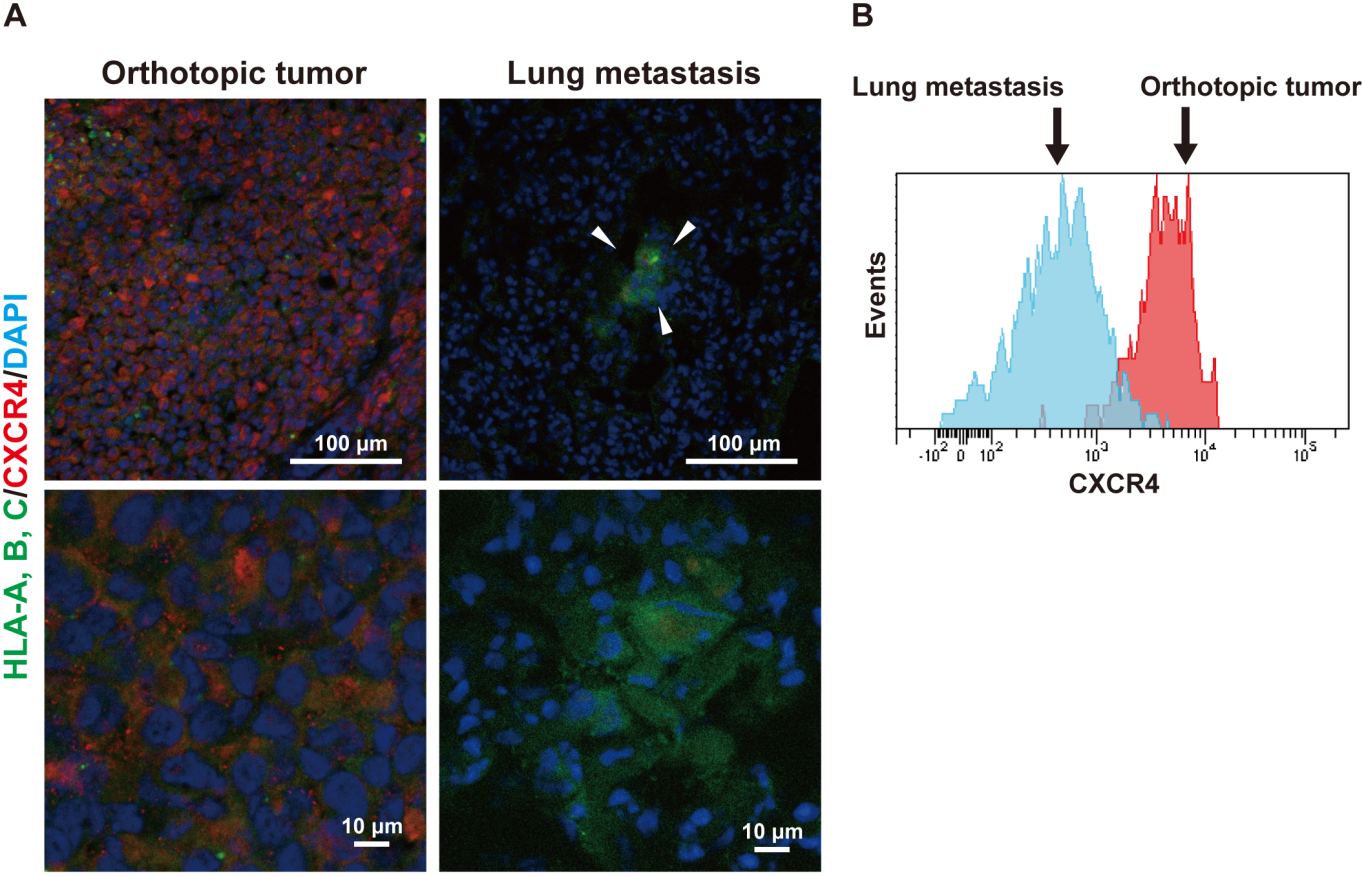
Downregulation of CXCR4 in metastasized dormant breast cancer cells. (A) Immunofluorescent images for CXCR4 and MDA-MB-231 breast cancer cells in the orthotopic tumor and metastatic lesions in the lung. Arrowheads indicate the metastatic tumor lesion in the lung. Green: human leukocyte antigen (HLA)-A, B, C; red: CXCR4; blue: nucleus. Scale bars: 100 μm for the low power field; 10 μm for the high power field. Representative images are shown. (B) Flow cytometric analysis of the expression level of cell surface CXCR4 of MDA-MB-231 breast cancer cells in the orthotopic tumors and metastatic lesions in the lung. The histogram with red indicates cancer cells in the orthotopic tumor, and the one with blue indicates metastasized cancer cells in the lung. Representative histograms are shown.

Next, we evaluated the expression level of CXCR4 in the metastasized cancer cells in the lung that were more prone to be in the dormant state than the cancer cells in the orthotopic tumor. Immunohistological examination of the expression level of CXCR4 in the PDX and cell line-derived tumor models was performed using fluorescent microscopy. In the cell line-derived tumor model, the signal for CXCR4 was much weaker in metastasized cancer cells in the lung than that in the cancer cells of the orthotopic tumors that were strongly stained with the anti-CXCR4 mAb (Fig 3A). Essentially the same results were obtained in the PDX model (S3 Fig). In the flow cytometric analysis, the signal for cell surface CXCR4 of the metastasized cancer cells was remarkably reduced compared with that of the cancer cells in the orthotopic tumors (Fig 3B). These findings suggest that CXCR4 is downregulated in metastasized cancer cells depending on the change of the environment that induces dormancy to cancer cells.

### Evasion of the metastasized cancer cells in the lung from the CXCR4/CXCL12 signaling

**Fig 4:**
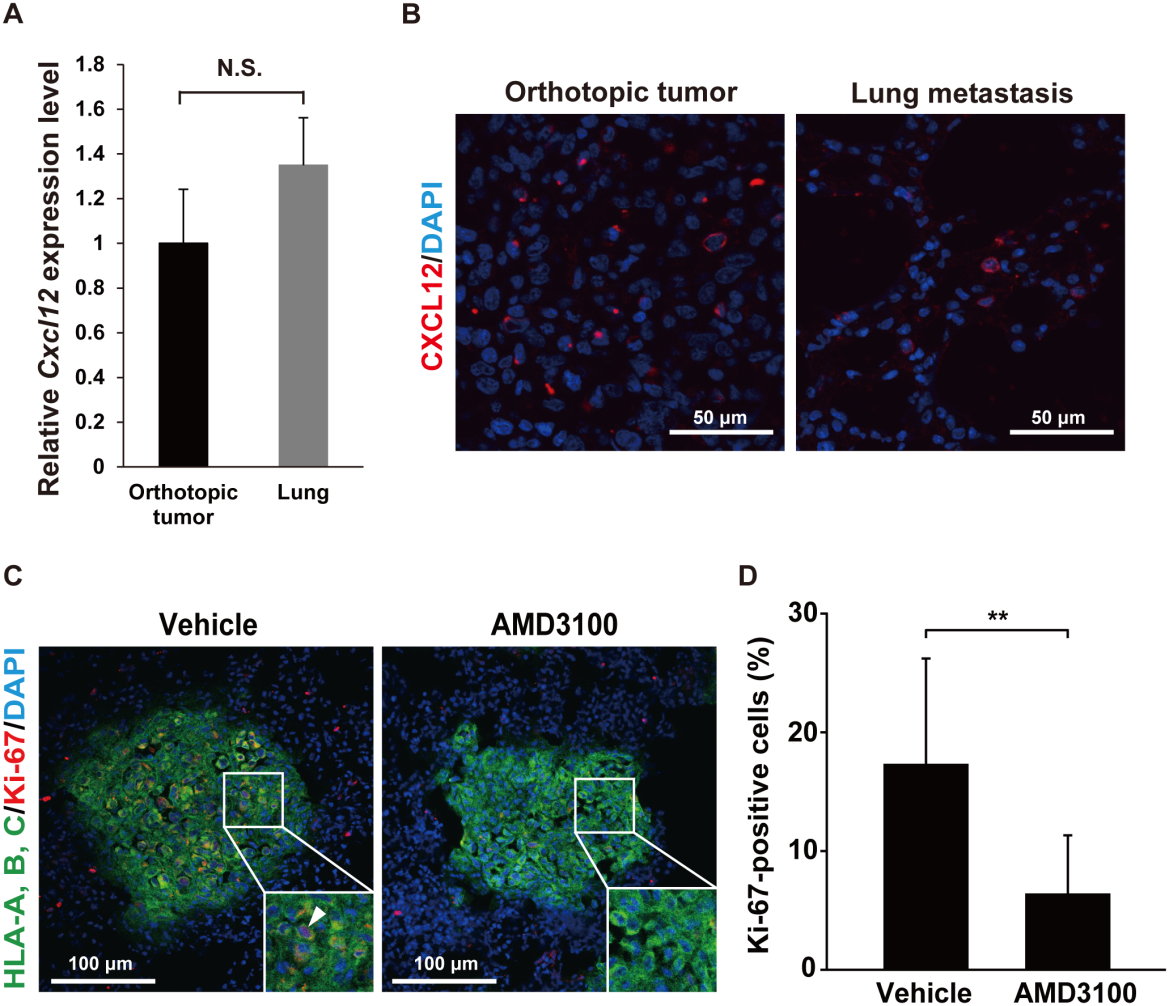
Enhancement of the dormancy of the cancer cells in the lung by the inhibition of CXCR4. (A) Quantification of the expression levels of the *Cxcl12* mRNA in the orthotopic tumor and the lung tissues by real-time quantitative reverse transcription PCR. The expression levels of the mRNA were normalized to those of *Gapdh* as an internal standard (orthotopic tumor: n = 5; lung: n = 4). No significant difference was observed between these two tissues. (B) Immunofluorescent images for CXCL12 in the orthotopic tumor and the metastatic lesions in the lung. The anti-CXCL12 mAb recognized both mouse and human molecules. The immunofluorescence signal for human leukocyte antigen (HLA)-A,-B,-C was not detected under the fixation conditions used here, but it was observed under the different fixation conditions used in Fig 1B. Red: CXCL12; blue: nucleus. Scale bars: 50 μm. Representative images are shown. (C) Immunofluorescent images for Ki-67 in the metastatic lesions in the lung of the mice administered with or without AMD3100. Green: HLA-A, B, C; red: Ki-67; blue: nucleus. Scale bars: 100 μm. Representative images are shown. The area in the white boxes are magnified 2.5-fold in size and shown in the insets. The arrowheads indicate the Ki-67-positive metastatic cancer cells in the lung. (D) The rates of the Ki-67-positive MDA-MB-231 cells in the metastatic lesions in the lung. The number of the Ki-67-positive or negative cancer cells was manually counted in twelve high power fields, and the rate of the Ki-67-positive MDA-MB-231 cells were calculated. (** p<0.005).

Because the results mentioned above suggest that CXCR4 is implicated in breast cancer dormancy in both the PDX and cell line-derived tumor models, and the cancer cells in the cell line-derived tumor model grew faster and metastasized more efficiently than those in the PDX model, we hereafter examined using the MDA-MB-231 cell line-derived tumor model. In the course of tumor growth, the CXCR4 ligand CXCL12 is mainly produced by the stromal cells forming the tumor niche [23,24]. The CXCL12-induced CXCR4-mediated intracellular signaling enhances tumor growth, cancer cell survival, and metastasis [25]. To evaluate the difference of the expression levels of CXCL12 between the orthotopic tumor and the lung, we quantified the expression levels of *Cxcl12* mRNA. In real-time quantitative RT-PCR analysis, its expression level was not significantly different between the orthotopic tumor and the lung (Fig 4A). Consistently, the immunofluoresence signal for CXCL12 including both mouse and human molecules was observed in both the orthotopic tumor and the metastatic lesions in the lung (Fig 4B). Next, to evaluate the role of the CXCL12-CXCR4 signaling in tumor growth, the CXCR4 antagonist AMD3100 [26] was repeatedly administered to the area adjacent to the MDA-MB-231 cell-derived tumors after the xenotransplantation. Consistent with previous reports [16,27,28], AMD3100 significantly suppressed the growth of orthotopic tumors in mice (S4 Fig). Then we analyzed the effect of the inhibition of the CXCR4 signaling on dormancy of the metastasized cancer cells in the lung. In this experiment, the tumor at the orthotopic site was surgically removed 6 week after the transplantation of MDA-MB-231 cells, when the tumor metastasized to the lung. One week after the surgical removal, AMD3100 was administered by daily subcutaneous injection for two weeks. The number of the Ki-67-positive cells among the metastasized cancer cells in the lung was significantly reduced by the administration of AMD3100 (Fig 4C and 4D). These findings suggest that the CXCL12-CXCR4 signaling is required for promoting tumor growth in this model, and that the cancer cells in which CXCR4 is downregulated are presumably less affected by this signaling to become dormant. Moreover, the reduction of the expression level of CXCR4 in the metastasized cancer cells is not dependent on the expression level of CXCL12 in the lung tissue around the metastasized cancer cells.

### Dynamic change of the expression level of CXCR4 depending on the change of tumor environment

**Fig 5:**
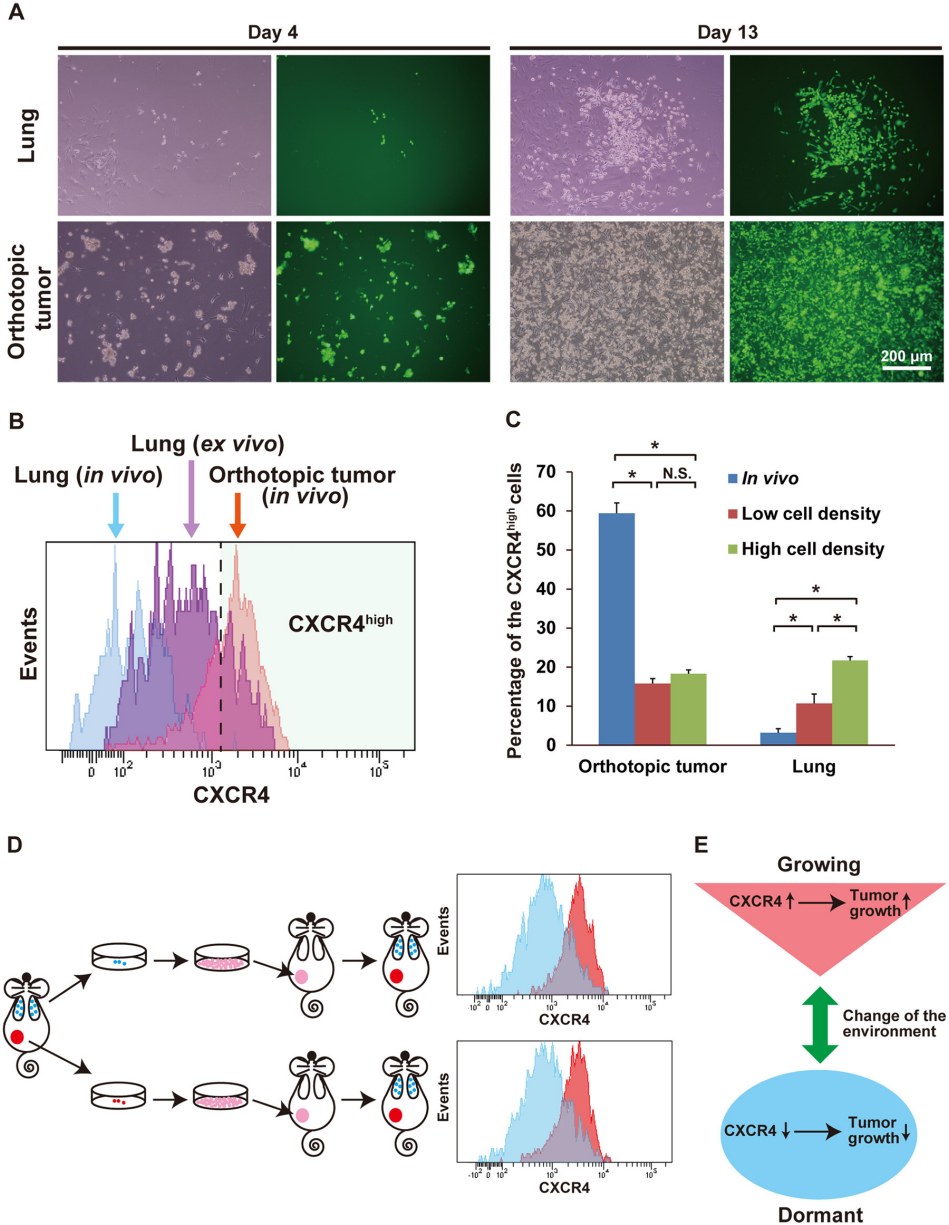
Dynamic change of the expression level of CXCR4 responding to the change of tumor environment. (A) Microscopic examination of cells obtained from the lung and orthotopic tumor in the culture conditions. Images on the left show the bright field view and images on the right show the fluorescent view. Green cells in the fluorescence view indicate breast cancer cells. Cells with no fluorescence around the cancer cells are the non-cancer cells. Scale bar: 200 μm. Representative images are shown. (B) Flow cytometric analysis of CXCR4 of the breast cancer cells obtained from the lung tissues in the culture condition. The histogram with blue color indicates cancer cells in the lung *in vivo*, purple indicates *ex vivo* cultured cancer cells obtained from the lung, and red indicates cancer cells in the orthotopic tumor *in vivo*. Representative histograms are shown. Cells in the light green colored area are cells with a high expression level of cell surface CXCR4. (C) Percentage of the *ex vivo* cultured cancer cells with a high expression level of cell surface CXCR4 in the low (70–80% confluent) and high (100% confluent) cell densities. At least three analyses were performed at each time point. Asterisks indicate the significant difference between the two groups. (D) The schema and histograms of the xenotransplantation experiment of the *ex vivo* cultured cancer cells originally obtained from the orthotopic tumor and the lung. Histograms show the expression level of cell surface CXCR4 of the cancer cells obtained from the orthotopic tumor and the lung of the mice that were xenotransplanted with the *ex vivo* cultured cancer cells originally obtained from the orthotopic tumor or the lung. The red colored histograms indicate the cancer cells obtained from the orthotopic tumors, and blue colored histograms indicate the cancer cells obtained from the lung. Representative histograms are shown. (E) Summary of our findings. The change of the tumor environment causes the change of the expression level of CXCR4. This change facilitated the tumor growth.

To evaluate whether the metastasized dormant cancer cells retain the ability to revert its growth and change the expression level of CXCR4 depending on the change of the environment around the cancer cells, we cultured dissociated lung cells of the cell line-derived tumor model, which included a small fraction of the metastasized cancer cells. Standard culture conditions, which allowed intensive cell proliferation of the parental MDA-MB-231 cells, were applied, and in addition, the cells obtained from the orthotopic tumors were also cultured in the same condition. In a few days, the cancer cells obtained from both the lung and the orthotopic tumor gradually started to proliferate, and the culture dish became confluent in about 9–13 days (Fig 5A). No significant difference was observed in the proliferation rate between the cancer cells obtained from the lung and the orthotopic tumor, although the proliferation rate of the cancer cells obtained from the lung was higher than the cells from the orthotopic tumors (S5 Fig). These findings suggest that the dormant cancer cells in the lung maintain the ability to regrow and restart proliferation as the environment changes to conditions that promote their proliferation.

Next we evaluated the expression level of cell surface CXCR4 in the *ex vivo* cultured cancer cells obtained from the cell line-derived xenograft tumor, and analyzed the percentage of cells that expressed a high level of CXCR4 by flow cytometry (Fig 5B and 5C). The expression level of CXCR4 was much higher in the orthotopic tumor than in the metastasized cancer cells *in vivo* (Figs 3, 5B and 5C). In the *ex vivo* culture, the expression levels of CXCR4 in the orthotopic cancer cells were markedly reduced in both low (70–80% confluent) and high (100% confluent) cell densities (Fig 5C). In contrast, the expression level of CXCR4 was markedly increased in the *ex vivo* cultured cancer cells obtained from the lung, and eventually reached almost the same level as that of the cells from the orthotopic tumors (Fig 5C). We observed that a long period of culture time and an increase of cell density were required for the cells from the lung tissues to maximize the expression level of CXCR4 (Fig 5C). These findings indicate that the change of the expression level of CXCR4 is a reversible phenomenon caused by the change of environment around the cancer cells; it is not, as presumed, an irreversible process caused by the genetic mutation of the cancer cells. In addition, these findings also indicate that the increase of the expression level of CXCR4 requires a certain amount of time and optimal cancer cell density.

To validate these findings, we xenotransplanted the *ex vivo* cultured cancer cells that were originally obtained from both the orthotopic tumor and the lung of the cell line-derived xenograft tumor-bearing mice. After 6 weeks of the transplantation, the xenotransplanted cancer cells originally obtained from both the orthotopic tumor and the lung repopulated the cells with a high expression level of CXCR4 in the orthotopic tumor and the cells with a low expression level of CXCR4 in the lung metastasis (Fig 5D). These findings indicate that the expression level of CXCR4 in cancer cells change depending on the change of the environment caused by local tumor development and cancer metastasis. Furthermore, this change facilitates or restrains the proliferation of the cancer cells, a feature highly associated with cancer malignancy and dormancy (Fig 5E).

## Discussion

In this study, we found that CXCR4 is not uniformly expressed in the tumor and in the course of the disease. The expression level of CXCR4 dynamically changes depending on the change of the tumor environment caused by tumor development or metastasis. Moreover, this change enhances or restrains tumor growth, and implicates in the proliferating or dormant phenotype of the cancer cells. A previous study showed that the dynamic change of the expression level of CXCR4 in Ewing sarcoma cells depends on the change of cellular stresses, such as growth factor deprivation, hypoxia, and space constraints [29]. Another report showed that the growth and metastasis of ovarian cancer cells expressing CXCR4 under suboptimal culture conditions were stimulated by the ligand CXCL12 [30]. In this study, we microscopically observed a remarkably high density of the cancer cells in the orthotopic tumors, which showed a high expression level of CXCR4, in contrast to the cancer cells in the micro-metastatic lesions in the lung that showed low cell numbers in the tissues and a low expression level of CXCR4 (Figs 1 and 3, S2B and S3 Figs). In addition, the area that was occupied by the vasculature was far lower in the orthotopic tumors than that of the lung tissues (Fig 1A). These findings suggest that the cancer cells in the orthotopic tumors are subjected to more severe cellular stresses, such as hypoxia and low nutrients, than the cells in the micro-metastatic lesions in the lung. In addition, the expression level of CXCR4 was increased in the dormant cancer cells by the *ex vivo* culture, and the induction required a long period of culture duration and a high increase of the cell density (Fig 5C). These findings suggest that the proliferation of the cancer cells and the increase of tumor amount themselves induce the suboptimal environment for the cells to maintain proliferating and metastatic states with a high expression level of CXCR4. The finding that the cancer cells in the *ex vivo* culture expressed a far lower level of CXCR4 than the cells in the orthotopic tumor *in vivo* (Fig 5C) suggests that stress-promoting factors other than a long period of time of culture and a highly increased cell density are needed for cells to express a higher expression level of CXCR4. Conversely, this hypothesis suggests that the dormant cancer cells in the micro-metastatic lesions in the lung are under a less stress-promoting environment than the cells in the tumor of the origin because of the lower tumor amount and easier access to the vasculatures in the tissues. Under this condition, cancer cells putatively have less need to compete with other cancer cells for nutrients and oxygen. Analysis of the metabolism of the cancer cells in the micro-metastatic lesions may help to evaluate this hypothesis.

In this study, the metastatic cancer cells showed a lower expression level of CXCR4 than the cells in the orthotopic tumors. In contrast, other reports showed relatively a high expression level of CXCR4 in the metastatic cancer cells in the tumor models generated by allotransplantation and xenotransplantation of cancer cells in mice [16,31]. Moreover, this report showed the requirement of time to induce the expression of CXCR4 in the metastatic tissues [16]. In these reports, the metastatic lesions in the organs were generated by tail vein injection of the cancer cells that formed macroscopically visible large lesions [16], or by a long period of incubation time (14 weeks) after the xenotransplantation of MDA-MB-231 cancer cells [31]. In contrast, we used the spontaneous metastatic model, which represented an extremely early stage of metastasis as compared with previous reports. In our model, the metastatic lesions were invisible with a macroscopic view of the lung, and flow cytometric analysis showed that the metastatic cancer cells accounted for approximately 1% or less of the total lung cells (S1 and S2A Figs). The difference of the expression level of CXCR4 in the metastatic cancer cells in our study and in previous reports may be caused by the different tumor amount and incubation period, as described above.

Although our findings do not sufficiently explain the mechanisms of the induction and exit of tumor dormancy, our findings suggest that CXCR4 facilitates tumor growth and serves as the switch of the tumor state from dormant to growing. Our present results are consistent with the previous results that the inhibition of the CXCL12-induced CXCR4-mediated signaling suppressed the growth of both primary and metastatic tumors [16,27,28]. In addition, we showed here that the inhibition of CXCR4 further enhanced the dormancy of the metastasized cancer cells in the lung. However, blocking of the CXCL12-induced CXCR4-mediated signaling was not able to completely suppress tumor growth or eliminate all cancer cells [16,27,28]. These findings suggest that the CXCL12-induced CXCR4-mediated signaling is not the indispensable survival factor or essential inducer of cancer cell growth. CXCR7 is another receptor for CXCL12 with rather high binding affinity [32,33] and is reported to be involved in cancer cell proliferation in a CXCR4-independent and/or-dependent manner [33]. Thus, the role of the CXCL12-CXCR7 signaling in the regulation of cancer cell dormancy will be studied in detail in future.

Finally, our finding that dormant cancer cells express a low level of CXCR4 suggests that treatments targeting the CXCL12-induced CXCR4-mediated signaling may be ineffective for cells at this stage. Similarly, anticancer agents that target the machinery of growing cells may not be effective for the dormant cancer cells, which are not actively growing. On the other hand, previous reports suggested that anticancer agents induce the expression of CXCR4 in cancer cells [34,35]. Cancer therapy using an anti-VEGF Ab upregulated CXCR4 in rectal cancer cells [34], and upregulation of CXCR4 and increased invasiveness mediated by reactive oxygen species, NF-κB, and HIF-1α was observed in pancreatic cancer cells treated by the chemotherapeutic agent gemcitabine [35]. These findings suggest that cancer cells switch from the dormant state with a reduced expression level of CXCR4 to the growing and metastatic state with an increased expression level of CXCR4 in response to increased hypoxic and oxidative stresses caused by the anticancer treatments. We may need to consider a combined therapeutic strategy that maintains the dormant cancer cells as dormant as well as decreases the proliferating activity of growing cancer cells.

## Supporting Information

S1 Fig Flow cytometric analysis of metastasized MDA-MB-231 cells in the lung. Flow cytometric analysis of the orthotopic tumor and the lung in the cell line-derived xenograft tumor model. The mouse that was xenotransplanted parental MDA-MB-231 cells that do not express ZsGreen1 was analyzed as a control (upper row). The particles were sorted and checked by microscopy whether they contained the cancer cells or the debris of the tumor. The particles, which showed a moderate level of ZsGreen1 and contained much of debris of the tumor, were excluded. 7AAD, 7-amino-actinomycin D.
(TIF)

S2 Fig Metastasis and dormancy of breast cancer cells in the patient-derived xenograft (PDX) model. (A) Flow cytometric analysis of the orthotopic tumor and the lung in the PDX model. The lung of the mouse that was not xenotransplanted was analyzed as a control (upper row). The particles were sorted and checked by microscopy whether they contained the cancer cells or the debris of the tumor. The particles, which showed a moderate level of human leukocyte antigen (HLA)-A, B, C and contained much of debris of the tumor, were excluded. 7AAD, 7-amino-actinomycin D. (B) Immunofluorescent images for Ki-67 in the orthotopic tumor and metastatic lesions in the lung in the PDX model. The arrowheads indicate the metastatic tumor lesions in the lung. Green: HLA-A, B, C; red: Ki-67; blue: nucleus. Scale bars: 100 μm for the low power field; 10 μm for the high power field. Representative images are shown.
(TIF)

S3 Fig Downregulation of CXCR4 in metastasized breast cancer cells in the patient-derived xenograft (PDX) model. Immunofluorescent images for CXCR4 in the orthotopic tumor and metastatic lesions in the lung of the PDX model. Arrowheads indicate the metastatic tumor lesion in the lung. Green: human leukocyte antigen (HLA)-A, B, C; red: CXCR4; blue: nucleus. Scale bars: 10 μm. Representative images are shown.
(TIF)

S4 Fig Suppression of the growth of the orthotopic tumors by AMD3100. Growth curves of the vehicle- or AMD3100-treated MDA-MB-231-derived orthotopic breast cancer xenograft tumors in mice (vehicle group: n = 5; AMD3100 group: n = 4). The final volume of the tumors in each group was significantly different (* p<0.05).
(TIF)

S5 Fig Proliferation rate of the *ex vivo* cultured cancer cells obtained from the orthotopic tumor and the lung. The number of the cancer cells in the culture dish at Day 0 and Day 7 of the *ex vivo* culture was examined using flow cytometry, and the ratio between them was calculated as a proliferation rate of the cells (n = 3). The difference of the proliferation rate between cancer cells obtained from the orthotopic tumor and the lung was not statistically significant.
(TIF)
