# Supplementary figures and images for: Downregulation of CXCR4 in Metastasized Breast Cancer Cells and Implication in Their Dormancy
Source: PLoS One. 2015 Jun 17;10(6):e0130032. doi: 10.1371/journal.pone.0130032 (PMC4470829; doi:10.1371/journal.pone.0130032)

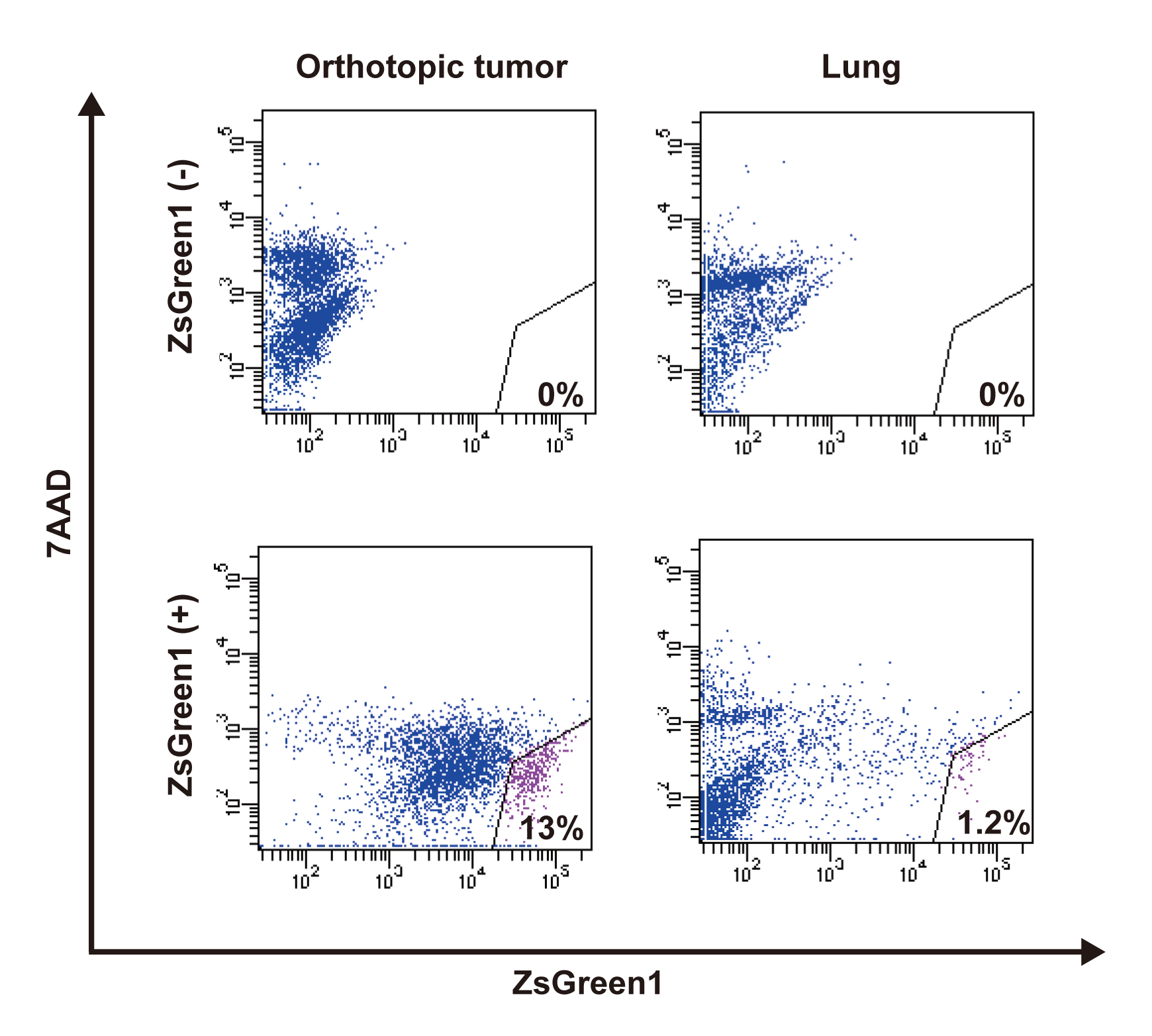

Supplement: S1 Fig — Flow cytometric analysis of the orthotopic tumor and the lung in the cell line-derived xenograft tumor model. The mouse that was xenotransplanted parental MDA-MB-231 cells that do not express ZsGreen1 was analyzed as a control (upper row). The particles were sorted and checked by microscopy whether they contained the cancer cells or the debris of the tumor. The particles, which showed a moderate level of ZsGreen1 and contained much of debris of the tumor, were excluded. 7AAD, 7-amino-actinomycin D. (TIF) [file pone.0130032.s001.tif]

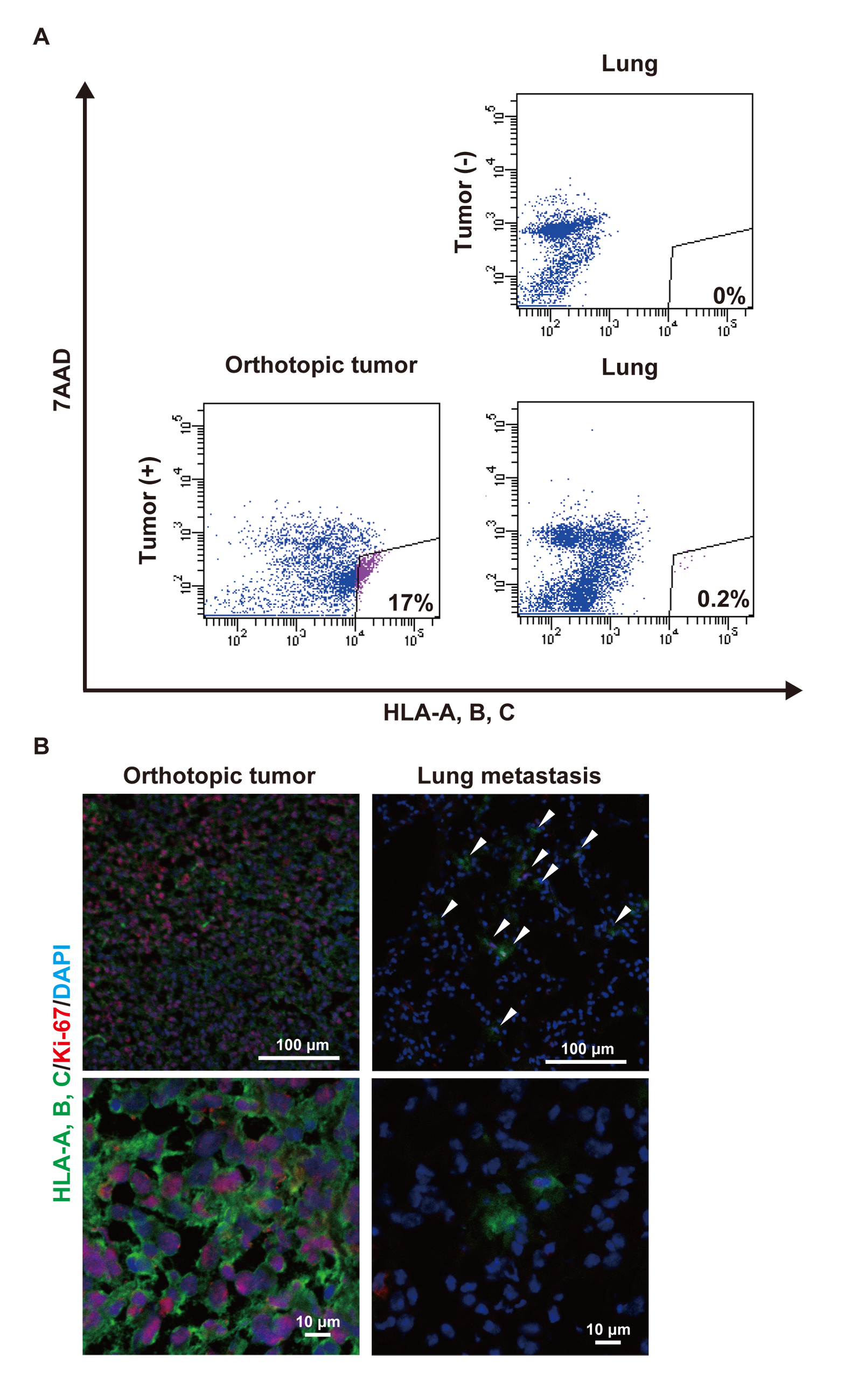

Supplement: S2 Fig — (A) Flow cytometric analysis of the orthotopic tumor and the lung in the PDX model. The lung of the mouse that was not xenotransplanted was analyzed as a control (upper row). The particles were sorted and checked by microscopy whether they contained the cancer cells or the debris of the tumor. The particles, which showed a moderate level of human leukocyte antigen (HLA)-A, B, C and contained much of debris of the tumor, were excluded. 7AAD, 7-amino-actinomycin D. (B) Immunofluorescent images for Ki-67 in the orthotopic tumor and metastatic lesions in the lung in the PDX model. The arrowheads indicate the metastatic tumor lesions in the lung. Green: HLA-A, B, C; red: Ki-67; blue: nucleus. Scale bars: 100 μm for the low power field; 10 μm for the high power field. Representative images are shown. (TIF) [file pone.0130032.s002.tif]

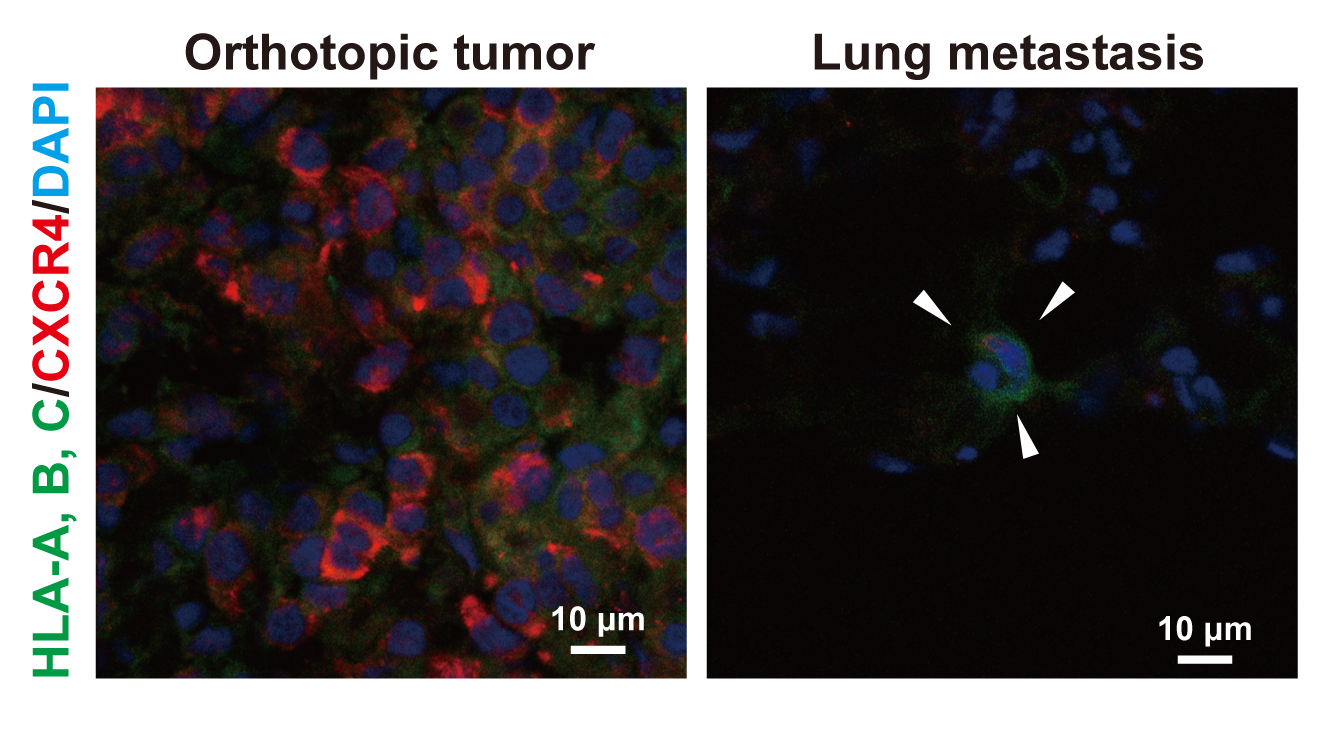

Supplement: S3 Fig — Immunofluorescent images for CXCR4 in the orthotopic tumor and metastatic lesions in the lung of the PDX model. Arrowheads indicate the metastatic tumor lesion in the lung. Green: human leukocyte antigen (HLA)-A, B, C; red: CXCR4; blue: nucleus. Scale bars: 10 μm. Representative images are shown. (TIF) [file pone.0130032.s003.tif]

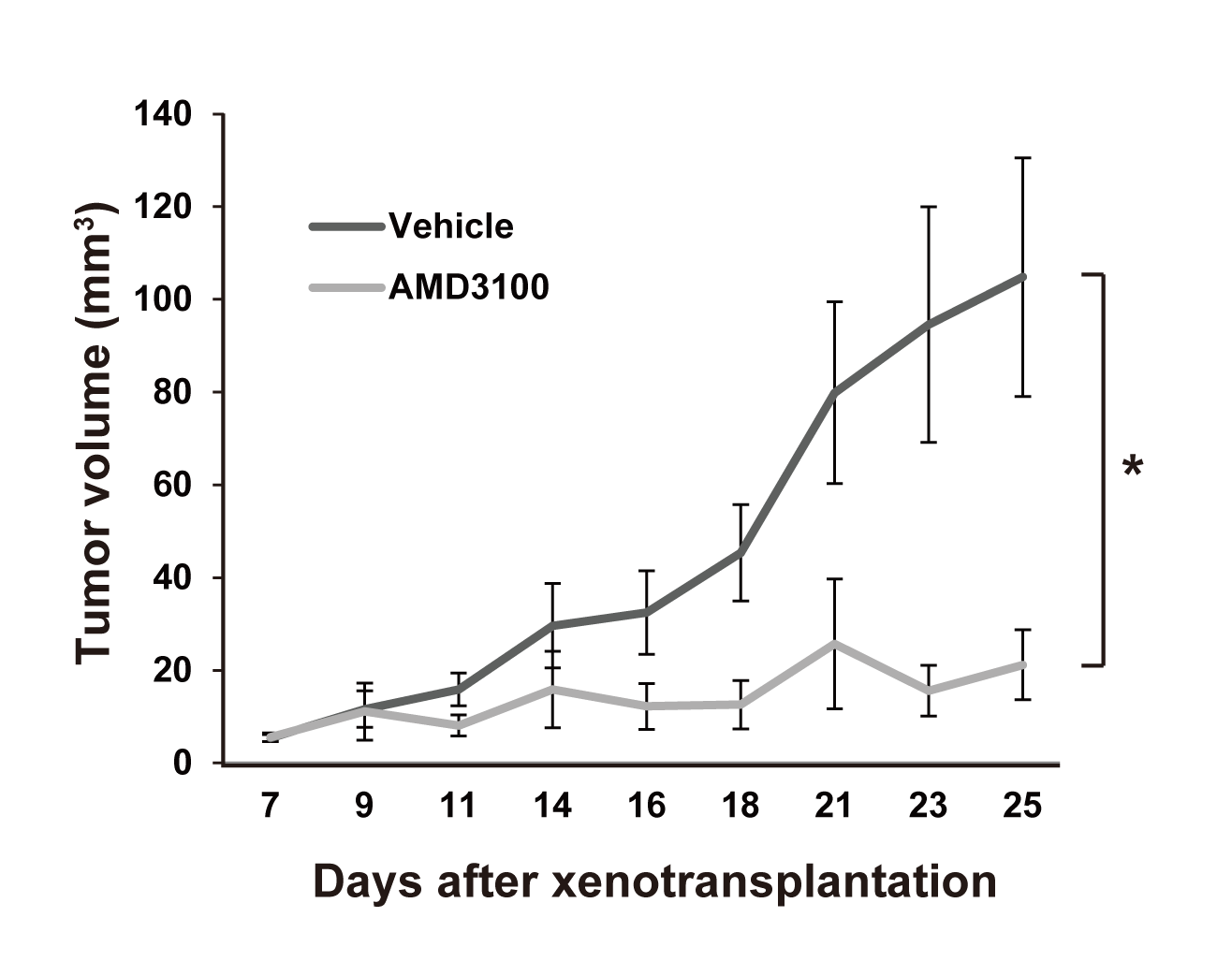

Supplement: S4 Fig — Growth curves of the vehicle- or AMD3100-treated MDA-MB-231-derived orthotopic breast cancer xenograft tumors in mice (vehicle group: n = 5; AMD3100 group: n = 4). The final volume of the tumors in each group was significantly different (* p<0.05). (TIF) [file pone.0130032.s004.tif]

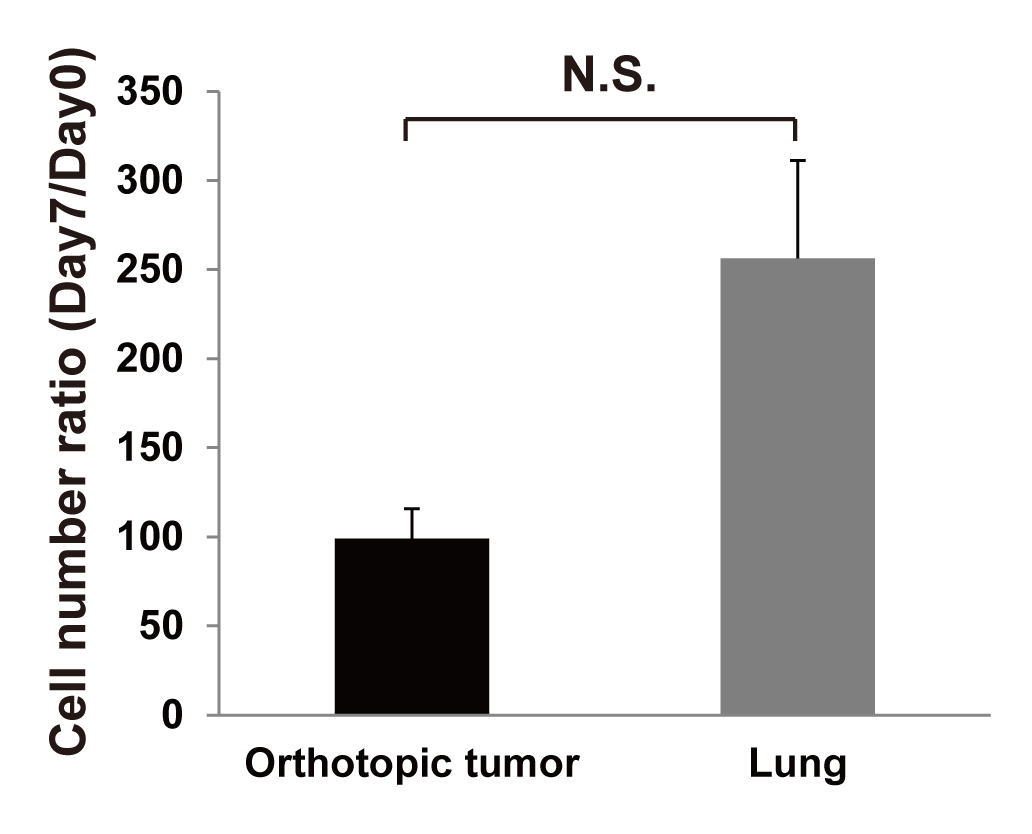

Supplement: S5 Fig — The number of the cancer cells in the culture dish at Day 0 and Day 7 of the ex vivo culture was examined using flow cytometry, and the ratio between them was calculated as a proliferation rate of the cells (n = 3). The difference of the proliferation rate between cancer cells obtained from the orthotopic tumor and the lung was not statistically significant. (TIF) [file pone.0130032.s005.tif]
